# Genome-wide identification, expression analysis, and functional study of the *bZIP* transcription factor family and its response to hormone treatments in pea (*Pisum sativum* L.)

**DOI:** 10.1186/s12864-023-09793-5

**Published:** 2023-11-22

**Authors:** Xiaozong Wu, Changhe Cheng, Rui Ma, Jianbo Xu, Congcong Ma, Yutao Zhu, Yanyan Ren

**Affiliations:** 1 Zhengzhou University of Light Industry, Zhengzhou, 450002 People’s Republic of China; 2 China Tobacco Zhejiang Industrial Co., LTD, Hangzhou, 310000 People’s Republic of China; 3 State Key Laboratory of Crop Stress Biology for Arid Areas, College of Plant Protection, Northwest A&F University, Yangling, 712100 Shaanxi People’s Republic of China; 4 College of Medical Technology, Luoyang Polytechnic, Luoyang, 471000 China; 5 College of Life Science and Engineering, Henan University of Urban Construction, Pingdingshan, 462500 China; 6 Henan University of Urban Construction, Pingdingshan, 467036 Henan China

**Keywords:** Pea, *bZIP* gene family, Evolution, Hormones

## Abstract

**Background:**

Basic leucine zipper (*bZIP*) protein is a plant-specific transcription factor involved in various biological processes, including light signaling, seed maturation, flower development, cell elongation, seed accumulation protein, and abiotic and biological stress responses. However, little is known about the pea *bZIP* family.

**Results:**

In this study, we identified 87 *bZIP* genes in pea, named *PsbZIP1* ~ *PsbZIP87*, via homology analysis using *Arabidopsis*. The genes were divided into 12 subfamilies and distributed unevenly in 7 pea chromosomes. *PsbZIPs* in the same subfamily contained similar intron/exon organization and motif composition. 1 tandem repeat event and 12 segmental duplication events regulated the expansion of the *PsbZIP* gene family. To better understand the evolution of the *PsbZIP* gene family, we conducted collinearity analysis using *Arabidopsis thaliana, Oryza sativa Japonica, Fagopyrum tataricum, Solanum lycopersicum, Vitis vinifera, and Brachypodium distachyon a*s the related species of pea. In addition, interactions between PsbZIP proteins and promoters containing hormone- and stress-responsive cis-acting elements suggest that the regulation of PsbZIP expression was complex. We also evaluated the expression patterns of *bZIP* genes in different tissues and at different fruit development stages, all while subjecting them to five hormonal treatments.

**Conclusion:**

These results provide a deeper understanding of *PsbZIP* gene family evolution and resources for the molecular breeding of pea. The findings suggested that *PsbZIP* genes, specifically *PSbZIP49*, play key roles in the development of peas and their response to various hormones.

**Supplementary Information:**

The online version contains supplementary material available at 10.1186/s12864-023-09793-5.

## Background

Pea (*Pisum sativum* L., 2n = 2x = 14) is a climbing herb native to West Asia, the Mediterranean, and Ethiopia. As a dual-purpose vegetable crop, pea exhibits cold and drought tolerance and is widely distributed in the world with strong adaptability [1, 2]. Pea is rich in vitamins, mineral elements, protein, and carbohydrates and is a protein source worthy of research and development. In addition, peas can promote nitrogen accumulation in soil and improve the physical properties of the soil, with a high utilization value [1, 3].

Transcription factors (TFs) are a group of cis-elements that specifically bind a specific sequence (promoter region) upstream of eukaryotic genes to regulate their expression [4]. Transcription factors are involved in many physiological processes, such as inducing the expression of related genes in response to changes in external stimuli and regulating plant growth and development, stress responses, and injury defense [5, 6]. The basic leucine zipper (bZIP) proteins are a large transcription factor family. The bZIP members have a conserved domain consisting of 60 to 80 amino acid residues which is further divided into the C-terminal alkaline amino acid domain and the N-terminal leucine zipper domain. The N-terminal has about 16 to 20 basic conserved amino acid residues, which can specifically recognize the ACGT sequence. The C-terminal is less conserved and composed of one or more heptapeptide repeat regions forming an α helix [7–9]. *bZIP* transcription factor plays an important role in substance accumulation and stress response, and regulating plant growth and development, stress responses and plant hormone signals [10–13].

Many *bZIP* gene families have been identified in many plant species, including *Arabidopsis thaliana* (75) [13], *switchgrass* (178) [14], *Oryza sativa* (89) [15], *Zea mays* (125) [16], *Fagopyrum tataricum* (96) [17], *Solanum lycopersicum* (69) [18], *Brachypodium distachyo* (96) [19] *Vitis vinifera* (55) [20], *Brassica napus* (247) [21], and *castor bean* (49) [22]. Previous studies demonstrated that *bZIP* transcription factors are involved in many important physiological processes, such as hormonal and sugar signaling [23, 24], seed maturation and germination [25], light responses [26, 27], salt and drought tolerance [28, 29] and pathogen defense [30, 31]. So far, *bZIP* gene family has mainly focused on abasic acid (ABA), as an important plant hormone, which is involved in the regulation of gene expression and related physiological processes in abiotic stress response [23, 24]. Meanwhile, the functions of the *bZIP* gene family have been studied in various plants. For example, in *A. thaliana*, more than half of the group A *bZIP* TFs (*AtbZIP39*/*ABI5*, *AtbZIP36*/*ABF2*/*AREB1*, *AtbZIP38*/*ABF4*/*AREB2*, *AtbZIP66*/*AREB3*, *AtbZIP40*/*GBF4*, *AtbZIP35*/*ABF1* and *AtbZIP37*/*ABF3*) have been intensively studied, most of these TFS play a central role in ABA and stress signaling [32, 33]. AtbZIP11 promoted the transcription of IAA3/SHY2, a negative regulator of root growth and development. IAA3/SHY2 inhibited auxin transport but promoted the expression of the Pin-formed (PIN) gene family to prevent auxin transportation to the apical meristem and inhibit root development [34]. ABF (ABA-Responsive element binding factors) transcription factors belonging to subfamily A of bZIP play an important role in abscisic acid (ABA) and stress responses. Overexpressing pepper CabZIP25 in *A. thaliana* and *wheat* Ta*bZIP*15 in *wheat* can improve the salt stress of crops [35, 36].

In this study, we identified 87 *bZIP* genes and performed a comparative analysis of their basic structure, motif composition, chromosomal localization, and gene duplication using recently published pea genomes. In order to further explore the development mechanism between different species, the pea *bZIP* gene was compared with six related genera and the evolutionary relationship between different species was analyzed. Finally, qRT-PCR was used to analyze the expression patterns of bZIP genes in different tissues and different stages of fruit development under five different hormone treatments, and some important candidate genes were selected. The results showed that the expression patterns of *bZIP* gene were different in different tissues of pea, which initially confirmed its biological significance in pea. In addition, our results lay the foundation for subsequent functional analyses of *bZIP* gene families in other species and provide a theoretical framework for further research in this field.

## Results

### Identification of the *bZIP* gene in pea

**Fig. 1:**
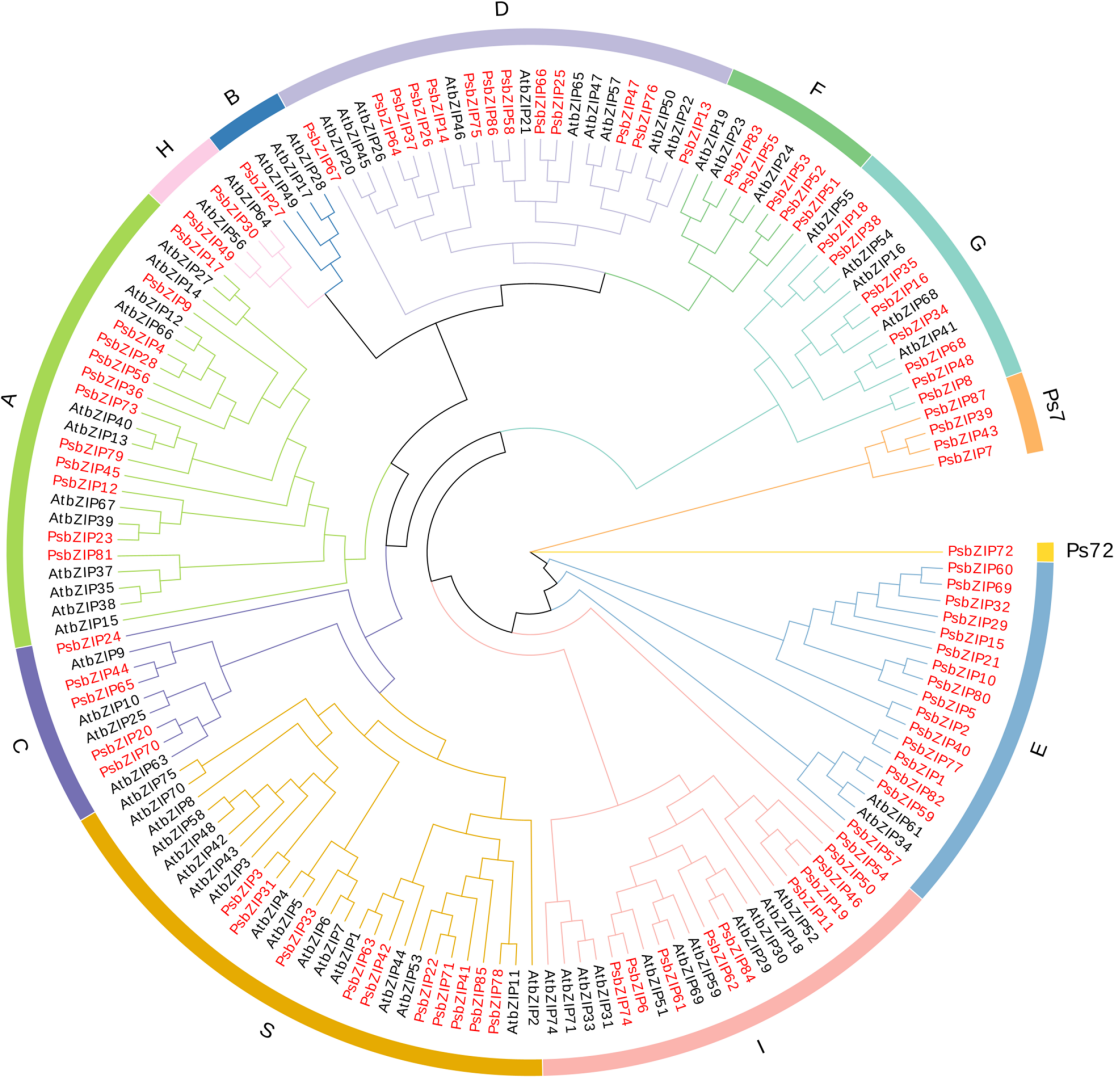
A phylogenetic tree of pea and *A. thaliana* basic leucine zipper (bZIP) proteins (twelve subfamilies). Red and black colors represent pea and *A. thaliana* proteins, respectively

We identified 87 *bZIP* genes, namely *bZIP1*-*bZIP87*, which were mapped to different chromosomes. Among the 87 bZIP proteins, bZIP22 protein had the least amino acids (144), while bZIP87 protein had the most amino acids (844). The molecular weight of the proteins ranged from 15.986 kDa (bZIP22) ~ 92.565 kDa (bZIP7), with a pI ranging between 4.65 (bZIP1) ~ 9.68 (bZIP87) with an average of 7.02. All *bZIP* genes contained bZIP DNA binding domains. Subcellular localization results showed that all *bZIP* genes were located in the nucleus, with 34 presenting in the cytoplasm, 21 in the chloroplast, 16 in the plasmid, 6 in the peroxisome, and 5 in the extracellular (Fig. 1; Supplementary Table 1).

### Multiple sequence alignment, phylogenetic analysis, and classification of *bZIP* genes

To analyze the phylogenetic relationships of pea bZIP proteins, we used MEGA 7.0 software to construct a phylogenetic tree of pea (87 *bZIPs*) and *Arabidopsis* (50 *AtbZIPs*) *bZIP* genes. The 87 *PsbZIP* genes were divided into twelve branches (groups 1–12) in the phylogenetic tree, according to the previously proposed Cenci and Rouard classification method and topology [37]. There was a consensus with the taxa of bZIP proteins in *Arabidopsis*, indicating that these *bZIP* genes remained stable during the evolutionary process.

Among the twelve subfamilies, subfamily E had the most members (16 *PsbZIP*), while subfamilies B and Ps72 had the fewest members (only 1 *PsbZIP*). All members were mostly concentrated in five subfamilies A, D, E, I, and S. The phylogenetic tree revealed that some of the *PsbZIPs* clustered closely with At*bZIPs* (bootstrap support ≥ 70), suggesting that these proteins might be homologous and have similar biological functions in pea and *A.thaliana* (Fig. 1; Supplementary Table 1).

**Fig. 2:**
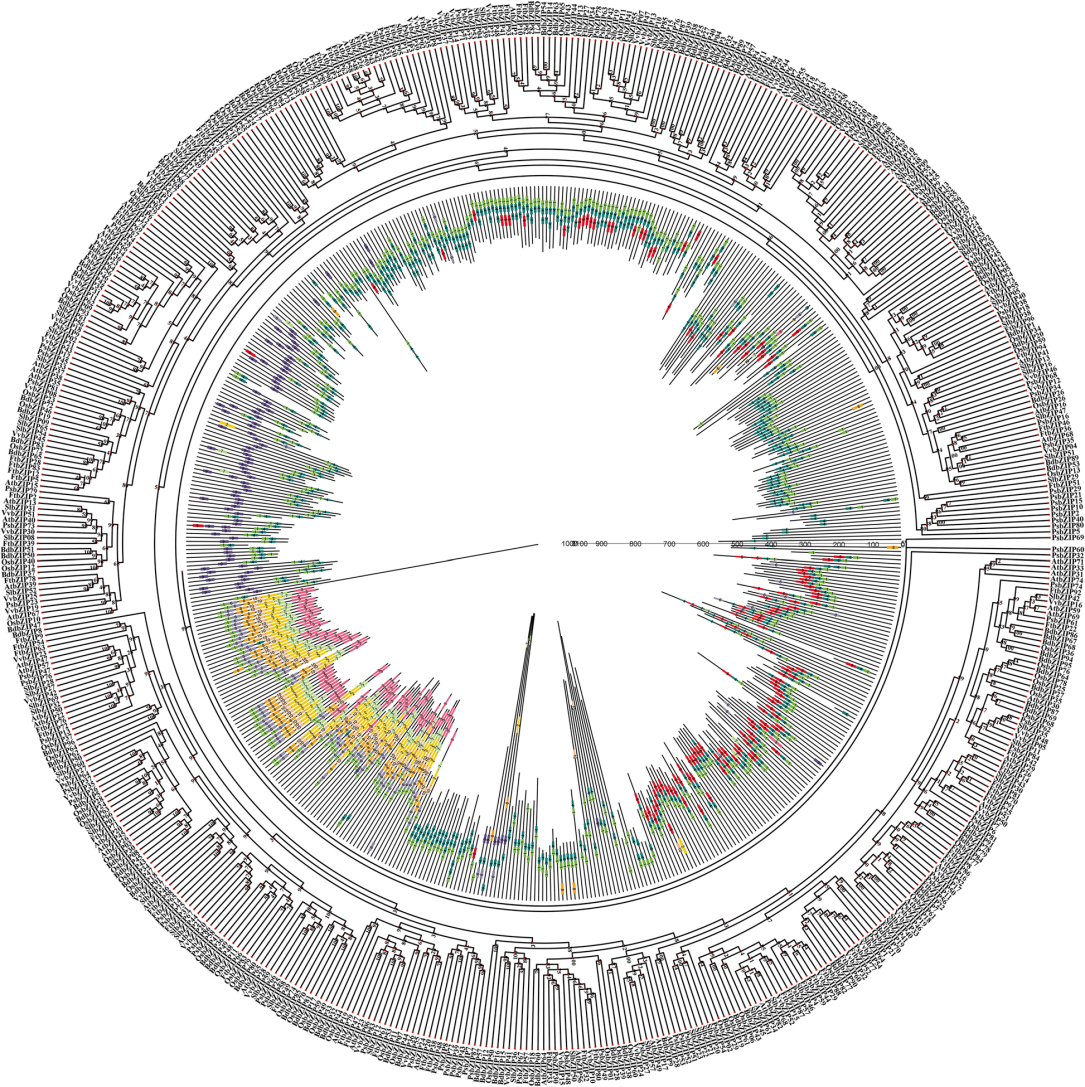
Phylogenetic relationships and motif compositions of the basic leucine zipper (bZIP) proteins of the seven different plant species (pea, *A. thaliana, V. vinifera, S. lycopersicum, O. sativa Japonica, F. tataricum, and B. distachyon*). **A** An unrooted phylogenetic tree was constructed using Geneious R11 via the neighbor-joining method. **B** Distribution of the conserved motifs in the bZIP proteins. The 10 boxes colored differently represent different motifs and their positions in each bZIP protein sequence

Four dicotyledons (*A. thaliana, O. sativa Japonica, F. tataricum, and S. lycopersicum*) and two monocotyledons (*V. vinifera and B. distachyon*) were selected to analyze the *bZIP* evolution of pea. The 87 identified *PsbZIP* genes were compared with the *bZIP* genes from 6 other plants containing 10 conserved motifs. As shown in Fig. 2, *PsbZIP* genes were unevenly distributed in the phylogenetic tree. These genes from the same subfamily tended to have the same themes and clustered together. Remarkably, almost all *bZIP* genes from these seven plants contained motif 1. Subfamily D contained the most motifs and showed diverse expressions, while subfamily E had only one motif (motif 4). In addition, many genes contained two motif 4, especially in subfamily G, and motif 4 and motif 1 were always closely linked. In summary, *PsbZIP* genes of the ps7 subfamily had a higher homology with the *bZIP* gene cluster of *T. buckwheat*. However, most of the *bZIP* genes of other groups had a high homology with the tomato *bZIP* gene cluster, indicating that they are more closely related and may have similar functions (Fig. 2; Supplementary Table 2).

### Conserved motif and structure analysis of *PsbZIP* genes

**Fig. 3:**
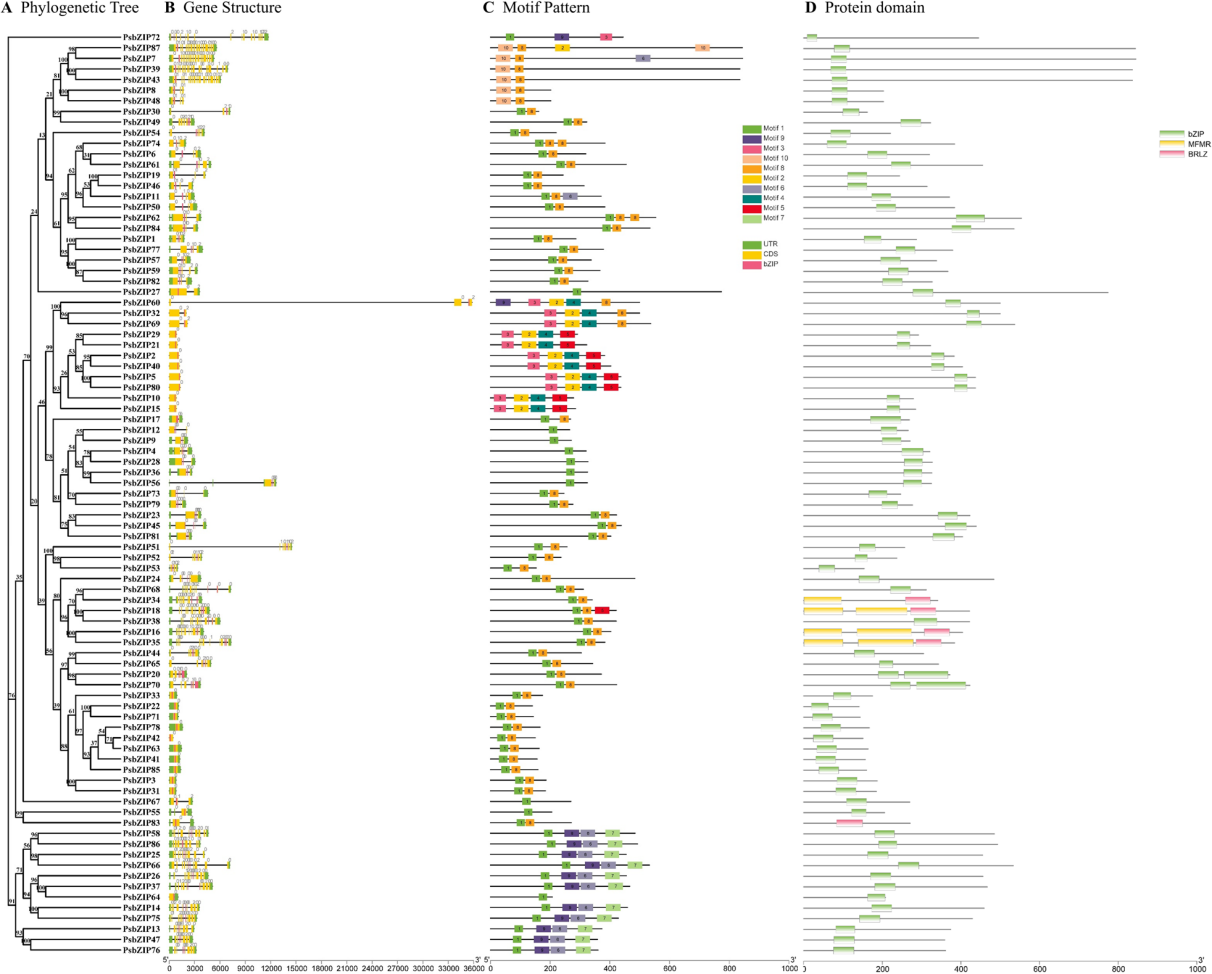
Analysis of conserved motifs, gene structures and protein domain in the phylogenetic tree of 87 *PsbZIP* genes. **A** A phylogenetic tree was constructed using the NJ method. **B** Exons and introns are shown as yellow rectangles and gray lines, respectively. The bZIP domain region is clearly marked. 0, 1, and 2 indicate exon phase. **C** Ten conserved motifs predicted in bZIP proteins are shown as differently colored boxes. **D** bZIP protein conserved domains are shown as green boxes

To study the structural diversity of the pea *bZIP* gene, we analyzed the location and number of exon–intron structures. 87 identified *PsbZIP* genes had varying numbers of exons, ranging from 1 to 18 (Fig. 3, Supplementary Table 2). The results showed that 18 (20.7%) of the 87 *PsbZIP* genes had no introns and were mostly concentrated in groups S and E. The number of introns ranged from 1 to 17 in the intron-containing *PsbZIP* genes. Additionally, subfamilies Ps7 and Ps72, containing the most *bZIP* genes, had the same intron and exon structures, with 18 exons and 17 introns. In general, *PsbZIP* genes of the same subfamily had similar gene structures. Subfamilies Ps7 and Ps72 exhibited greater structural differences in the number of introns and thus could have more functions.

To further evaluate the structural diversity of *PsbZIP* genes, we analyzed the motifs of *PsbZIP* genes using online motif software. We identified ten motifs in PsbZIP protein, named Motif 1 to Motif 10. Most of the genes contained Motif 1, and it is remarkable that subfamily A contained only one motif, while subfamily D contained the largest number of motifs. *PsbZIP* genes in the same subfamily often have similar motif composition. Further analysis showed that some motifs were distributed in specific positions. For example, motif 1 was always distributed at the beginning of the motif region, while motif 8 was distributed at the end. Sequence domain analysis of PsbZIP proteins revealed that almost all PsbZIP proteins contain bZIP conserved domains. In addition, PsbZIP16, PsbZIP18, PsbZIP34, PsbZIP35, from the same family of G, contained MFMR and BRLZ conservative domain structure. Overall, these results indicated that genes from the same subfamily have similar genetic composition and structures and tend to cluster together, in line with the phylogenetic tree's population classification.

### Chromosomal distribution and duplication of *PsbZIP* genes

**Fig. 4:**
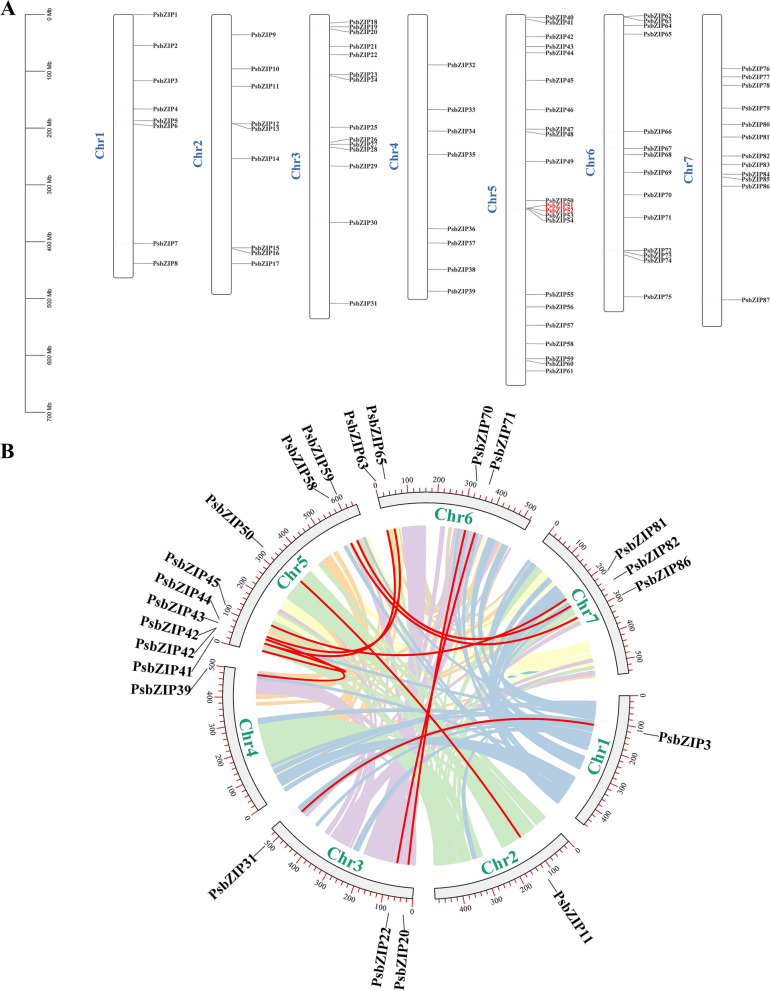
The chromosomal distribution and synteny blocks of the pea basic leucine zipper (*bZIP*) genes. **A** Distribution of the 87 *PsbZIP* genes on different chromosomes. The scale represents the length of chromosomes. Black bars indicate chromosomes. The chromosome number is displayed on the left side of each black bar. **B** Schematic representation of the chromosomal distribution and inter-chromosomal relationships of pea *bZIP* genes. Colored lines indicate all synteny blocks in the pea genome, and red lines indicate duplicated *bZIP* gene pairs

*bZIP* genes were physically mapped on chromosomes based on the newly published pea genome database. The 87 *bZIP* genes were distributed on seven chromosomes (Chr), and each *bZIP* gene was named according to its physical position on the chromosome (Fig. 4A; Supplementary Table 3). Chr5 contained the most *PsbZIP* genes (22–25.3%), followed by Chr3 and Chr6 (8–16.1%), while Chr1 and Chr4 contained the least *PsbZIP* genes (8–9.2%). Notably, *bZIP* genes were all evenly distributed on different chromosomes.

Gene duplication events, including tandem repeat events and segmental duplications, are essential in gene amplification and generating new gene functions [38]. The 200 kb range of chromosomal regions containing two or more genes is defined as tandem repeat events [39]. Accordingly, a duplication event analysis of the *bZIP* genes was performed to explore the evolutionary conservation of the gene family in peas. The results showed that the duplications of the *PsbZIP* gene family included both tandem and segmental duplication. As shown in Fig. 4B, *bZIP51* and *bZIP52* tandem repeat regions were located on Chr 5 of the pea genome. Of the seven linkage groups (LGs) in the pea genome, 12 pairs of *PsbZIP* gene fragments were localized on seven pea chromosomes, all located on two different LGs. These results suggested that some *PsbZIP* genes may have undergone fragment replication. These replication events were the main drivers of new functions in *PsbZIP* genes during evolution (Fig. 4B; Supplementary Table 4).

### Collinearity analysis of the *PsbZIP* and *bZIP* genes in different species

**Fig. 5:**
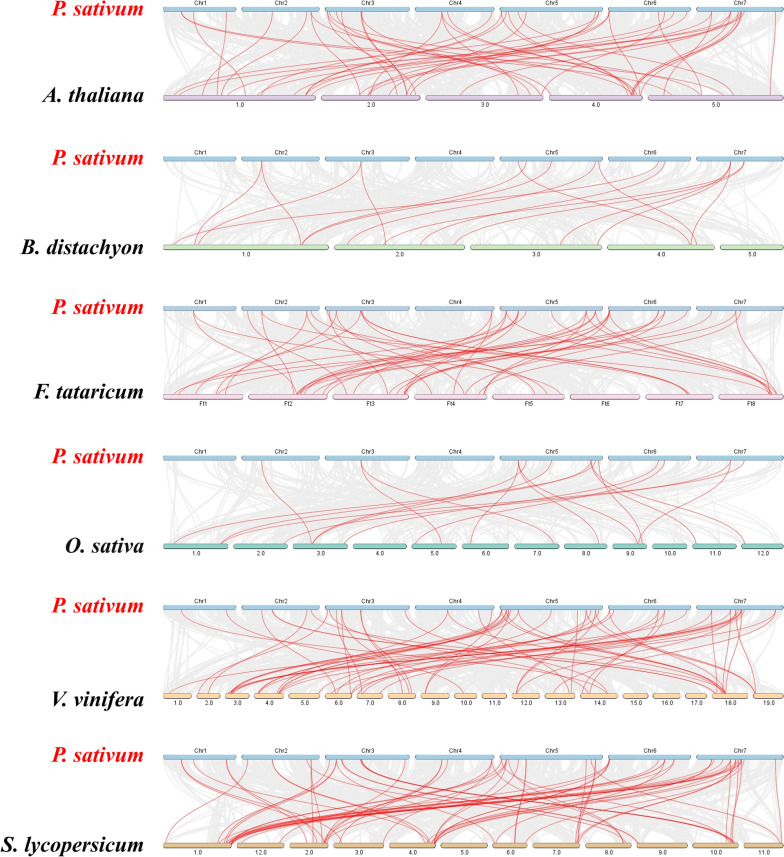
Analysis of basic leucine zipper (*bZIP*) genes between pea and six representative plant species (pea, *A. thaliana, V. vinifera, S. lycopersicum, O. sativa Japonica, F. tataricum, and B. distachyon*)*.* Gray lines in the background indicate the neighboring blocks in the genomes of peas and other plants, while the red lines represent the syntenic pea *bZIP* gene pairs

A homology map between peas and six representative species was constructed to explore the evolution of *bZIP* genes in peas. These species included four dicotyledons (*A. thaliana*, *V. vinifera*, *F. tataricum*, and *S. lycopersicum*) and two monocotyledons (*O. sativa* Japonica and *B. distachyon*). We found that 87 *PsbZIP* genes were colinear with those of *V. vinifera* (55), *S. lycopersicum* (69), *A. thaliana* (75), *O. sativa* Japonica (89), *F. tataricum* (96), and *B. distachyon* (96), and the number of homologous pairs among these species was 16, 18, 46, 49, 60 and 69, respectively (Fig. 5; Supplementary Table 5).

Homology analysis of these six plants revealed at least one pair of genes homologous to *PsbZIP*, such as *PsbZIP27*, *PsbZIP45*, *PsbZIP59*, *PsbZIP70*, and *PsbZIP71*, indicating that these homologous genes are highly conserved and may have existed prior to ancestral divergence. Additionally, these genes were speculated to have played an important role in the evolution of the *bZIP* gene family in peas. The collinearity analysis of these six species showed that *PsbZIP45* had the largest number of collinear genes (11), suggesting that these homologous gene pairs may have formed through gene replication during the differentiation of dicotyledonous and monocotyledonous plants (Fig. 5; Supplementary Table 5).

### Analysis of the cis-acting elements in *PsbZIP* promoters

**Fig. 6:**
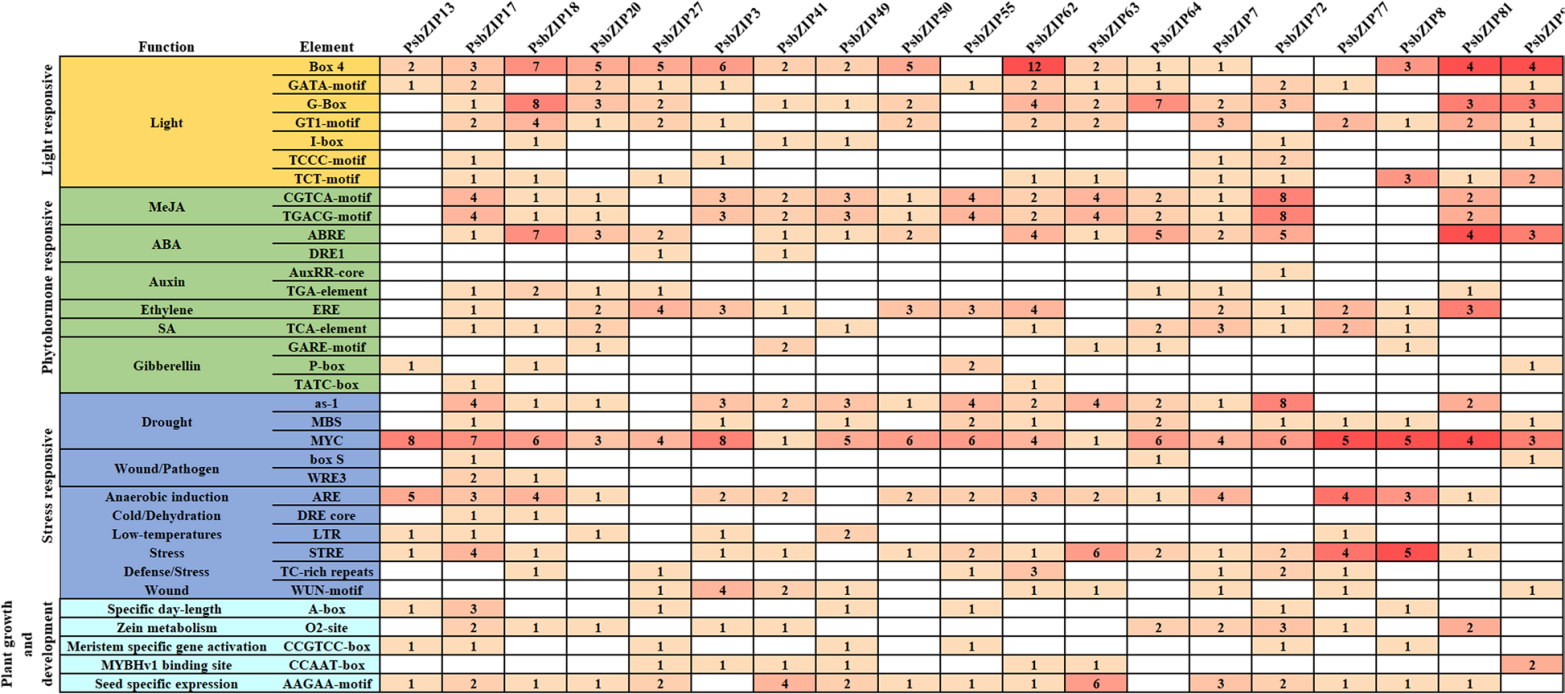
The distribution of cis-acting elements in promoters of pea basic leucine zipper (*PsbZIP*) gene family members

The promoter regions of *PsbZIP*s were analyzed to explore the tissue-specific expression and stress response patterns of these genes. The cis-acting elements in the promoter can be divided into four categories: light-responsive, hormone-responsive, stress-responsive, and plant growth and development-related elements. Individual *PsbZIP* genes in pea mostly contained the phytohormone response elements, including ABA response elements (ABRE) and MeJA hormone response elements (containing CGTCA- and TGACG-motifs). In addition, several MYC elements were found in all *PsbZIP* genes, suggesting that *bZIP* genes may be involved in drought resistance-related pathways. Thus, all *PsbZIP* genes contained drought (MYC) elements, while 73.7% of *PsbZIP* genes contained MeJA and ABA response elements (Fig. 6; Supplementary Table 6). The promoters of *PsbZIP17*, *PsbZIP18*, *PsbZIP20*, *PsbZIP41* and *PsbZIP64* contained reaction elements such as IAA -, ET -, SA-, ABA-, MeJA- and GA-. These results suggest that some cis-acting elements may regulate the expression of different tissues (seeds and meristem). In addition, we speculated that *PsbZIP* genes may be involved in tissue development and responses to hormonal and abiotic stress.

The promoter cis-elements and transcription factors can regulate the precise initiation and efficiency of transcription. We used PlantTFDB to explore the potential TFs binding *PsbZIP* promoter. The results showed that *PsbZIP72* had the most transcription factors while *PsbZIP27* had the least. However, all *PsbZIP* genes were regulated by several ERF and MYB TFs. Studies have shown that ERFs can regulate the expression of target genes via JA signaling to protect against *Boea chinensis* in *A. thaliana*, suggesting that *PsbZIP* may indirectly regulate JA synthesis against pathogens [40, 41]. It has also been reported that *AtMYB74* responds to osmotic stress, water deficit, and seed development by regulating ABA [42]. Therefore, it is speculated that *PsbZIP* may exert potential biotic and abiotic stress responses through ERF and MYB regulation.

**Fig. 7:**
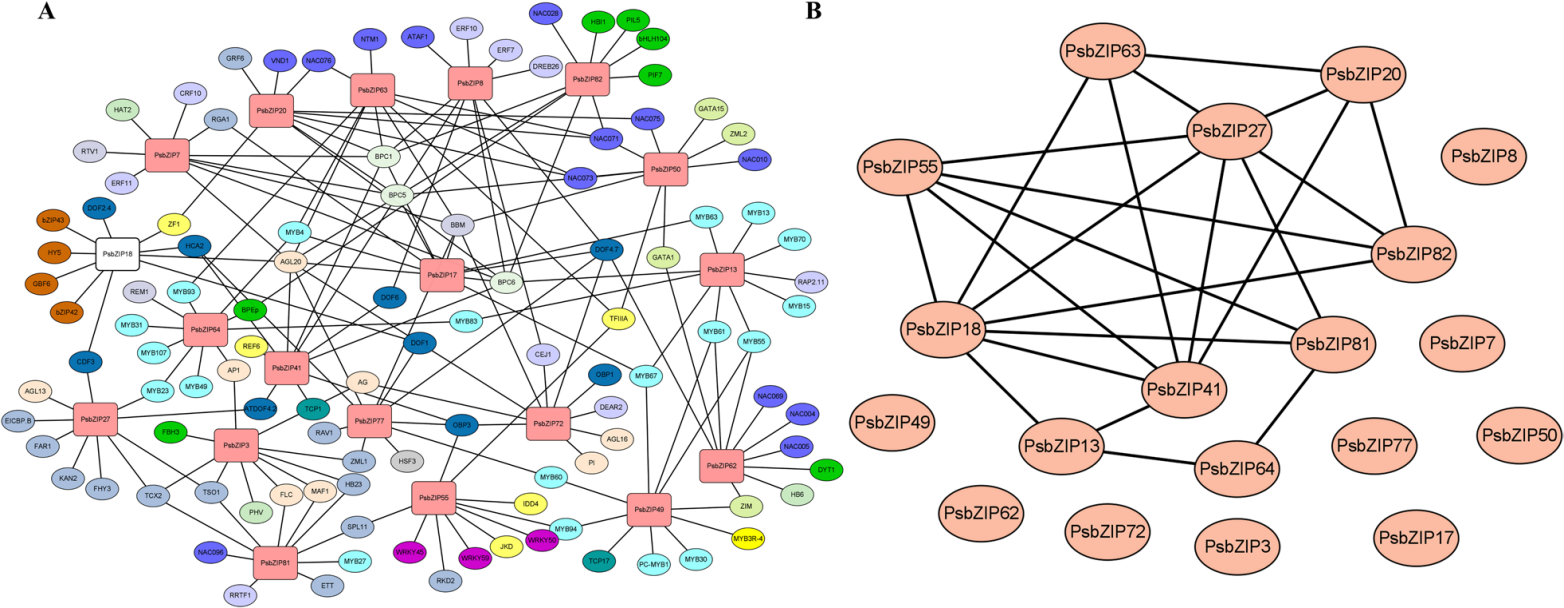
A The regulatory network between *PsbZIP*s and potential transcription factors (TFs). Red boxes represent *PsbZIP* genes, and the different colored oval structures represent different TFs. Light blue, dark blue, blue, and pink represent MYB, DOF, NAC, and *bZIP*, respectively. B A protein–protein interaction network of basic leucine zipper proteins of pea (PsbZIPs) based on their orthologs in *A. thaliana*

To better understand the *bZIP* gene regulatory mechanisms in pea, we used the most homologous *Arabidopsis* species as a basis for the reciprocal prediction of bZIP proteins in pea. As shown in Fig. 7, seven genes among the ten interacting *PsbZIP* members were *PsbZIP18* and *PsbZIP27*. Interestingly, *PsbZIP27* and *PsbZIP63* could simultaneously interact with *PsbZIP18*, *PsbZIP20*, and *PsbZIP41* and with each other. In *Medicago truncatula*, *bZIP17* and *bZIP60* TFs regulate the synthesis of JA and triterpenoid saponins [43]. GBF6 plays a role in seed color formation in oilseed rape by regulating the flavonoid biosynthesis pathway-related genes [44]. Thus, the homologous genes, *PsbZIP27* and *PsbZIP63*, may also be involved in the flavonoid synthesis pathway through hormonal regulation.

### Expression patterns of *PsbZIP* genes in different plant organs

**Fig. 8:**
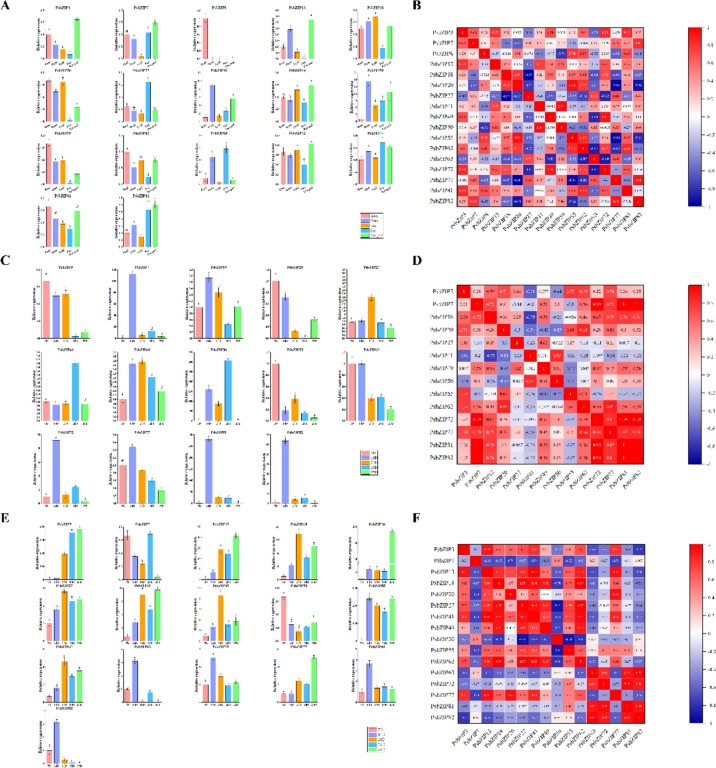
Tissue-specific gene expression of the 17 basic leucine zipper genes of pea (*PsbZIP*) at the different fruit development stages. Expression patterns of the 17 *PsbZIP* genes in pea fruit, leaf, root, stem, and pea pod tissues. Error bars represent the standard errors with three replications. Lowercase letters indicate significant differences among treatments (α = 0.05, LSD). B Positive number = positive correlation; negative number = negative correlation. Red numbers indicate a significant correlation at the 0.05 level. C Expression patterns of the 17 *PsbZIP* genes at 7, 14, 21, 28, and 35 days post-anthesis (DPA). Error bars represent the standard errors with three replications. Lowercase letters indicate significant differences among treatments (α = 0.05, LSD). D Positive number = positive correlation; negative number = negative correlation. Red numbers indicate a significant correlation at the 0.05 level

To further evaluate the potential functions of *PsbZIP* genes, we selected 12 subfamilies with a total of 17 representative genes to analyze the expression patterns in four plant organs (roots, stems, leaves, and flowers). The *PsbZIP* genes exhibited different expression patterns in root, stem, leaves, peas, and pea pods, suggesting that these genes may play diverse regulatory roles. All genes were expressed in different tissues (Fig. 8A, B). Two genes (*PsbZIP27* and *PsbZIP63*) had the highest expression in peas, five (*PsbZIP8*, *PsbZIP20*, *PsbZIP55*, *PsbZIP62*, and *PsbZIP81*) had the highest expression in root, while six (*PsbZIP3*, *PsbZIP7*, *PsbZIP13*, *PsbZIP49*, *PsbZIP72*, and *PsbZIP82*) had the highest expression in pea pods (*p* < 0.05). Most genes from the same subfamily had similar expression patterns, suggesting that these genes may have similar functions. We also found that all *bZIP* genes were least expressed in leaves, and thus speculate that *bZIP* genes may be more related to the development of roots, peas, and pea pods in pea plants.

Since the tissue-specific expression results showed that most *PsbZIP* genes were highly expressed in peas and pods, we speculated that *PsbZIP* might regulate the development of pea fruits. Therefore, we evaluated the effects of *PsbZIP* genes on the nutritional composition and development speed of peas and pods at different stages (Fig. 8C, D). We analyzed the expression of 14 *bZIP* genes at five different post-anthesis stages (7D, 14D, 21D, 28D, and 35D) to identify genes that could regulate pea fruiting-related genes. The results showed that most *PsbZIP* genes exhibited different expression patterns at these five stages of fruit development. The expression of three genes (*PsbZIP3*, *PsbZIP*20, *PsbZIP55*, and *PsbZIP62*) significantly decreased, while the expression of most genes (*PsbZIP7*, *PsbZIP50*, *PsbZIP72*, *PsbZIP81*, and *PsbZIP82*) increased expression with pea fruit development (*p* < 0.05). As shown in Fig. 8, most of the genes were upregulated in pea pods with increasing time, indicating that *bZIP* genes are positively regulated in pea pods (Fig. 8E, F). This also demonstrated that *bZIP* genes play an essential role in fruit development, providing a theoretical basis for studying the nutritional value of pea fruits.

### Expression patterns of *PsbZIP* genes under various hormonal treatments

**Fig. 9:**
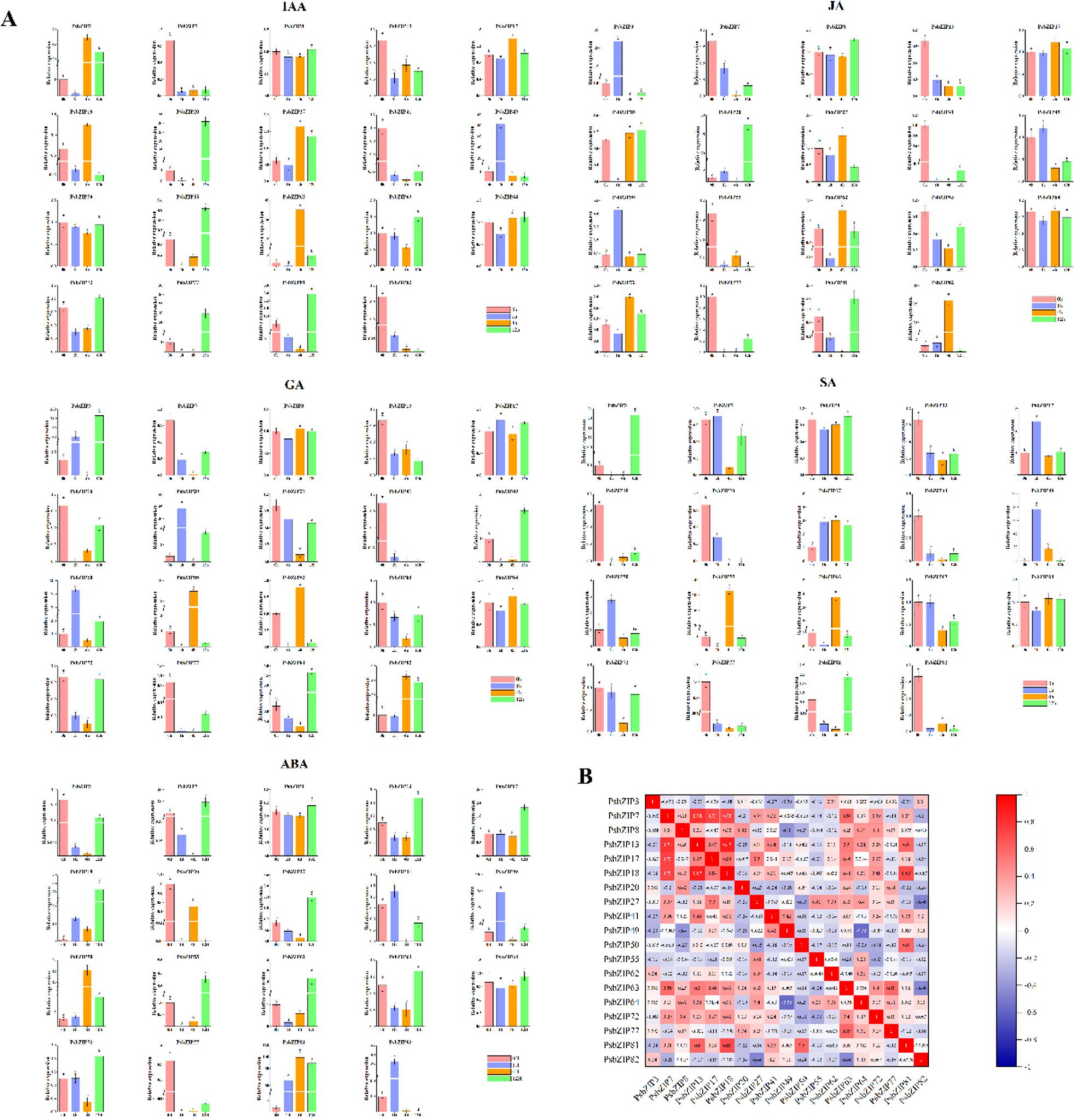
Expression analysis of the 19 basic leucine zipper genes of pea (*PsbZIP*) under different hormonal treatments (ABA, IAA, JA, SA, and GA3). **A** Expression analysis of the 19 *PsbZIP* genes. Error bars represent the standard error of three replicates. Lowercase letters above the error bars indicate significant differences among the treatments (α = 0.05, LSD). **B** Positive numbers = positive correlation; negative numbers = negative correlation. Red numbers indicate a significant correlation at the 0.05 level

To further determine whether the expression of *PsbZIP* genes is influenced by different hormones, we analyzed the expression of 19 representative *PsbZIP* genes under five hormonal treatments. The results showed that some *PsbZIP* genes exhibited significantly upregulated or downregulated expression patterns under different hormonal treatments. For example, almost half of the genes were upregulated after the ABA treatment, while *PsbZIP3* and *PsbZIP20* were down-regulated under JA treatment. The expression of *PsbZIP49* was the highest under IAA treatment, and the expression of most genes had the most significant responses at 12 h after treatment. Most genes showed a down-regulation trend under GA treatment, while *PsbZIP3*, *PsbZIP20*, *PsbZIP50*, *PsbZIP55*, and *PsbZIP82* showed an up-regulation trend (*p* < 0.05). Conversely, most genes showed a significant up-regulation under SA treatment. Notably, *PsbZIP49*, *PsbZIP62*, and *PsbZIP81* were highly expressed under all five hormonal treatments and could be further investigated as potential candidate genes (Fig. 9).

## Discussion

### Structural and evolutionary analyses of *PsbZIP* genes

Pea* (Pisum sativum* L., 2n = 2x = 14) is a mixed vegetable crop. Studies have shown that pea is rich in vitamins, minerals, proteins and carbohydrates, and it is a protein source worthy of research and development. bZIP TFs are involved in various plant biological processes, including light signaling, seed maturation, flower development, cell elongation, seed accumulation protein, and abiotic and biological stress responses [45–50]. *bZIP* TFs also play a crucial role in the growth and development of pea. The rapid development of genome sequencing in recent years has contributed to the identification and characterization of *bZIP* genes in many plants, including *V. vinifera* (55), *S. lycopersicum* (69), *A. thaliana* (75), *O. sativa* Japonica (89), *F. tataricum* (96), and *B. distachyon* (96). However, to date, the *PsbZIP* family is still poorly understood. Terefore, in this study we identifed and performed a preliminary functional test of *bZIP* gene family members in pea.

In this study, we identified 87 *bZIP* genes in pea, which encoded proteins with varying lengths between 141 and 845 amino acids. A comparative genomic analysis of the gene structure revealed that *bZIP* genes contained different numbers of introns, ranging from 0 to 17. Most of the coding proteins (except for subfamilies E and S) show structural complexity and variability, which may be attributed to gene duplication during evolution. Almost all PsbZIP proteins contain the bZIP conserved domain. PsbZIP16, PsbZIP18, PsbZIP34, PsbZIP35 were derived from the same subfamily G and contained both MFMR and BRLZ conserved domains, suggesting that subfamily G might have different functions than other subfamilies. In general, introns may increase the length of genes and the frequency of intergenic recombination, and alter their regulatory role [51]. Genes without introns may be conserved during species evolution [52–54]. Genes from the same subfamily have similar motif composition and number of introns. Therefore, we speculate that they may share a common evolutionary origin and molecular function, which can also be used to predict the function of unknown proteins.

The identified *bZIP* genes were divided into twelve subfamilies based on the conserved structural domains of *Arabidopsis*, and each group contained at least one *bZIP* gene from *Arabidopsis* and pea (except ps7 and ps72). This suggested that these genes were conserved during evolution and may have some biological functions (Fig. 1). Gene amplification is the main driver for generating new functional genes during evolution and can be divided into segmental duplications and tandem replication [55]. Compared to segmental replication, tandem duplication events occupy a larger proportion of plant genomes, with an approximately 10% incidence in *Arabidopsis* and *rice* [56, 57]. We found more bZIP proteins in pea compared with *V. vinifera* (55), *A. thaliana* (75), and *S. lycopersic* (69), indicating that there are likely more gene duplication events in pea. These duplication events can generate new functional genes to help plants adapt to harsh environments [58]. The mapping results showed that 87 *PsbZIP* genes were unevenly distributed on 7 chromosomes of pea (Fig. 3A). Furthermore, homology analysis of *PsbZIP* genes showed that one pair of tandem duplication and twelve pairs of fragment duplicates were present in pea (Fig. 3B). These homologous genes located on different chromosomes may have promoted the evolution and diversification of *PsbZIP* genes, resulting in more *bZIP* genes in pea than in other dicotyledon plants(*A. thaliana*, *V. vinifera* and *S. lycopersicum*).

To further explore the developmental mechanisms of *bZIP* genes, we generated six comparative syngeneic maps showing the relationship between pea and other species (four dicotyledons and two monocotyledons). Among them, *PsbZIP* genes of ps7 and ps72 subfamilies have high homology with buckwheat *bZIP* gene cluster, and most *bZIP* genes of other groups, such as subfamily I and G, have high homology with tomato *bZIP* gene cluster. By analyzing the motif composition of the *bZIP* gene, we found that the *bZIP* gene contains 10 motifs, and different subfamilies contain similar motifs, while the D subfamily contains almost all motifs. These results suggest that genes in the same subfamily evolved more closely and may have similar functions. It is worth noting that, the homology analysis of these six plants revealed at least one pair of genes was homologous to *PsbZIP*, such as *PsbZIP27*, *PsbZIP45*, *PsbZIP59*, *PsbZIP70* and *PsbZIP71*. *PsbZIP45* had the highest collinearity numbers (11), suggesting that these homologous gene pairs may have formed through gene replication during the differentiation of dicotyledonous and monocotyledonous plants.

Promoter is an important cis-element of gene expression regulation, which can control the level of gene expression. Based on the analysis of bZIP gene promoters, we divided them into four categories: light-responsive, hormone-responsive, stress-responsive, and plant growth and development-related elements. All gene promoters contain myc elements, and we speculated that the *bZIP* gene family played an important role in drought stress. At the same time, most of the genes also contained a large number of hormone response elements. For example, *bZIP72* contained up to 16 response elements of MeJA, indicating that *bZIP72* may be involved in responding to JA signals. In the following qRT-PCR verification, it was also verified that *bZIP72* was significantly up-regulated under meja treatment. *bZIP18* has the largest number of ABA-responsive elements and shows a very high expression change under ABA treatment, further indicating that *bZIP18* may be involved in responding to ABA signals. It is very interesting that in the following protein interaction prediction, *bZIP18* can interact with 7 bZIP proteins, and we can take it as an important candidate gene for the following functional exploration.

### Expression patterns and function prediction of *PsbZIPs*

Gene expression analysis is often used as an essential step to provide useful clues for functional prediction [59]. In this study, the expression patterns of 17 genes, which were the representatives of twelve subfamilies, were explored in different tissues at different developmental stages. The results showed that almost all *bZIP* genes were significantly expressed (more than a twofold difference) in all tissues. *AtbZIP* TFs in the *Arabidopsis* C group exhibit important functions such as regulating fruit development [60]. A corresponding homologous gene, *PsbZIP20*, was down-regulated in pea fruits with growth, while its expression was upregulated in pods, suggesting that *PsbZIP20* may be more related to pod growth and development. Meanwhile, two genes (*PsbZIP27* and *PsbZIP63*) had the highest expression in pea fruits, five (*PsbZIP*8, *PsbZIP20*, *PsbZIP55*, *PsbZIP62*, and *PsbZIP81*) had the highest expression in roots, while six (*PsbZIP3*, *PsbZIP7*, *PsbZIP13*, *PsbZIP49*, *PsbZIP72*, and *PsbZIP82*) had the highest expression in pea pod (*p* < 0.05). Interestingly, most of the genes were most expressed at 14 days in peas, and almost half of the genes were most expressed at later pod stages. Notably, *PsbZIP81* was most significantly expressed in pea fruits, while *PsbZIP55* was most significantly expressed in pea pods, suggesting that they may be potential candidate genes affecting pea fruits and pods.

In addition, we examined the expression patterns of 19 genes from 12 subfamilies in different tissues under five hormone treatments. Our results showed that almost all *bZIP* genes were differentially expressed (i.e., showed more than a twofold difference) in different tissues in response to different hormonal treatments (*p* < 0.05). For example, most of the genes showed a trend of down-regulation under GA treatment, while they showed a trend of up-regulation under the remaining four hormones, showing a positive correlation. Most of the genes showed the most significant expression response at 12 h after treatment. It is worth noting that *PsbZIP49*, *PsbZIP62* and *PsbZIP81* were highly expressed under all five hormone treatments, which can be used as potential candidate genes for further study (Fig. 9).

Previous studies showed that ABA signaling plays an important role in the development and growth of plant tissues/organs such as fruits, flowers, roots, and seeds. It has also been reported that the bZIP protein is involved in ABA signaling [61, 62]. According to a study on *A. thaliana*, *bZIP*s from subgroup A (AtbZIP39, AtbZIP36, AtbZIP38, AtbZIP35, and AtbZIP37) play a major role in ABA signaling [10, 63, 64]. PsbZIP17 and PsbZIP81, belonging to the A subfamily, are highly similar to AtbZIPA2 and were significantly upregulated under ABA treatment. He Qing et al. found that soybean GmbZIP19 TF improved resistance to *Pseudomonas syringae* and *Sclerotinia* by upregulating the expression of abscisic acid, jasmonic acid, and salicylic acid induction genes [65]. The expression of the *GmbZIP15* gene improved the resistance of soybean plants to sclerotia and Phytophthora root rot. *GmbZIP15* activates the plant hormone signaling pathway by binding to the G-box element in the promoter region of plant hormone-related genes, thereby improving disease resistance [66]. The corresponding homologous gene, *PsbZIP55,* was upregulated under five hormonal treatments, indicating that *PsbZIP55* and *GmbZIP19* may have similar functions and could be candidate genes for disease resistance. Furthermore, *OsbZIP48* inhibited the expression of gibberellin synthesis-related protein, kaurene oxidase 2 (OsKO2), which is related to gibberellin synthesis, leading to plant stunting. A homologous gene, *PsbZIP49*, was also significantly downregulated under gibberellin treatment [67]. In conclusion, the results showed that these genes were significantly upregulated at different times during hormonal treatment. We hypothesized that the different expression patterns might be due to complex protein interactions coordinating the expression of multiple genes through a network of feedback mechanisms [68].

## Conclusion

In conclusion, we identified and comprehensively analyzed 87 *bZIP* genes in pea. The genes were classified into twelve subfamilies and were unevenly distributed across seven chromosomes. Genes within the same subfamily shared similar motifs and gene structures, suggesting their potential functional similarities. Moreover, both fragment and tandem repeats were identified as the primary driving forces for generating novel functions within the *PsbZIP* gene family. Particularly, fragment repeats appeared to significantly contribute to the evolution of pea *bZIP* genes. Notably, the promoters of almost all *PsbZIP* genes contained several hormone and stress response elements, with ERF being a key transcription factor involved in their regulation. Additionally, we conducted a structural analysis and evaluated expression patterns of the *bZIP* gene family in pea. Our findings highlighted the critical role of *PsbZIP49* in pea development, demonstrating its involvement in the development of pea fruits and pods and responses to hormonal stress.

## Materials and methods

### Gene identification

The whole pea genome was downloaded from the Ensembl website (http://ensemblgenomes.org), and *bZIP* gene family members were obtained based on two BLASTp approaches. First, all possible bZIP proteins were identified using BLASTp (score value ≥ 100, e value ≤ 1e-10) with the trihelix protein sequence of *Arabidopsis* as the reference [69]. Second, the PFAM protein family database (http://pfam.sanger.ac.uk) was used to produce a Hidden Markov Model (HMM) file with the *bZIP* domain [70], and then an HMM model cutoff value of 0.01 was applied to compare the bZIP protein sequences of pea (http://plants.ensembl.org/hmmer/index.html) in HMMER 3.0. The availability of the *bZIP* core sequence was confirmed using PFAM and SMART program (http://smart.emblheidelberg.de). We identified 87 *bZIP* genes which served as the initial sequences to confirm bZIP proteins (https://blast.ncbi.nlm.nih.gov/Blast.cgi? PROGRAM = blastp&PAGE_TYPE = BlastSear-ch&LINK_LOC = blasthome) via blastp [71, 72]. Finally, several characteristics of *bZIP* genes, such as the sequence length, isoelectric point (pi), molecular weight (MW), and subcellular localization, were identified using ExPasy. A 2000 bp sequence upstream of the start codon (ATG) of the *PsbZIP* gene was extracted from pea genome using TBtools, and its cis-acting elements were analyzed using PlantCare (http://bioinformatics.psb.ugent.be/webtools/plantcare/html). Thereafter, TFs were predicted using PlantTFDB [73] and visualized via Cytoscape [74].

### *bZIP* gene structure

The default parameters of ClustalW were used to create multiple protein sequence alignments based on the domain sequences of the characterized bZIP proteins of *A. thaliana*. The deduced amino acid sequences of *bZIP* domains from different subfamilies were manually regulated using GeneDoc software and MEGA7.0 [75]. The exon–intron structure of the *bZIP* gene was analyzed using the Gene Structure Di*bZIP*ay Server (http://gsds.cbi.pku.edu.cn) online program. MEME Online Applications (http://meme.nbcr.net/meme/intro.html) were then employed to identify the protein sequences using the following parameters: 6 ~ 200 optimum motif width and 10 maximum number of motifs [76]. The conserved domain of PsbZIP protein was analyzed by hmmscan and NCBI-CDD.

### Chromosomal distribution and gene duplication events

All *PsbZIP* genes were mapped on different pea chromosomes based on a physical map and visualized using the Circos program. The multiple collinear scanning toolkit (MCScanX) was then used (with default parameters) to analyze the replication events of *PsbZIP* genes [77]. Finally, the homology of *bZIP* genes between pea and six other plants (*O. sativa*, *A. thaliana*, *F. tataricum*, *S. lycopersicum*, *V. vinifera*, and *B. distachyon*) was determined using Dual Synteny Plotter (https://github.com/CJ-Chen/TBtools).

### Phylogenetic analysis and classification of *PsbZIP* gene family

All identified *PsbZIP* genes were first clustered into diverse groups based on the classification of *AtbZIP*s. A neighbor-joining (NJ) tree was built using the Jukes-Cantor model in MEGA 7.0. The phylogenetic tree was generated with a bootstrap value of 1000 assigned via the BLOSUM62 cost matrix in Geneious R11. Moreover, we generated a multi-species phylogenetic evolutionary tree that included all bZIP protein sequences from pea and the other six plant species (*O. sativa*, *A. thaliana*, *F. tataricum*, *S. lycopersicum*, *V. vinifera*, and *B. distachyon*). All protein sequences were downloaded from the UniProt database (https://www.uniprot.org). A protein–protein interaction analysis was performed using the STRING database (http://string-db.org) with PsbZIPs as queries and *A. thaliana* bZIP proteins as the reference. The promoter cis-acting elements were predicted using PlantCare and PlantTFDB.

### Plant materials, growth conditions, and different hormone treatments in pea

The pea seed used in the experiment was provided by Li Long of Agricultural University of Hebei. Zhongwan 6 is the variety we used. Pea plants were cultivated in pots containing a mixture of soil and vermiculite (1:1) in a growth room. The growth room was maintained at a temperature regime of 25 °C during the 16-h daytime period and 20 °C during the 8-h nighttime period. The relative humidity in the growth room was set at 75%. 45 days after planting, the leaves, roots, stems, peas, and pea pods were collected from five individual plants under the same growth environment. Fruit sampling was conducted when the first seed setting occurred, and subsequent samples were collected every other week for five consecutive harvests (7D, 14D, 21D, 28D, and 35D). The samples were immediately snap-frozen in liquid nitrogen and stored at -80 °C until further analysis. To determine the expression pattern of 19 *bZIP* genes under different hormones, we conducted hormonal stress treatments at the seedling stage (21 days after planting) using abscisic acid (ABA) (250 μmol/L), indole-3-acetic acid (IAA) (250 μmol/L), gibberellin A3 (GA3) (250 μmol/L), jasmonic acid (JA) (500 μmol/L), and salicylic acid (SA) (500 μmol/L). Each stress treatment was replicated five times, and qRT-PCR analysis was performed after sampling at 0 h, 1 h, 4 h, and 12 h, respectively.

### Total RNA extraction, cDNA reverse transcription, and qRT-PCR analysis

Total RNA was extracted from all samples using a plant RNA extraction kit (vazymes) following the manufacturer's instructions. Next, a cDNA library was constructed through reverse transcription of 1 mg RNA samples using 5 × HiScript® Reverse Transcriptase (vazymes) and 4 × gDNA (vazymes) kits in accordance with the manufacturer's protocol. The expression of some representative genes was then analyzed by qRT-PCR, with at least three biological replicates. The primers used were designed by Beacon Designer 7 (Supplementary Table 7). Relative mRNA expression was normalized to the actin gene (GADPH) mRNA expression, as the internal control, and was calculated using the 2^−(ΔΔCt)^ method [78].

### Statistical analyses

JMP6.0 (SAS Institute) was used to perform the analysis of variance (ANOVA) tests, and multiple comparison tests of ANOVA results were performed using the least significant difference (LSD) method at *p* < 0.05 and *p* < 0.01 significance levels. Histograms were generated using Origin version 8.0 (OriginLab, Northampton, MA, USA).

### Supplementary Information

**Additional file 1: Supplementary Table 1.** List of the 87 *PsbZIP* genes identified in this study.

**Additional file 2: Supplementary Table 2.** Analysis and distribution of conserved motifs in other plants *bZIP* proteins.

**Additional file 3: Supplementary Table 3.** The tandem duplication events of *PsbZIP*  genes.

**Additional file 4: Supplementary Table 4.** The 11 pairs of segmental duplicates in  Pisum sativum *bZIP* genes.

**Additional file 5: Supplementary Table 5.** One-to-one orthologous relationships between  Pisum sativum and Solanum lycopersicum.

**Additional file 6: Supplementary Table S6.** Cis-regulatory elements in the promoter region of *bZIP* genes.

**Additional file 7: Supplementary Table 7.** Primers of sequences.

## Data Availability

Whole genome sequence information for *Pea* was obtained from the Ensembl genome website (http://ensemblgenomes.org). The seed used in this experiment was Zhongwan 6. The datasets supporting the conclusions of this study are included in the article and in additional files.
